# Integrative Machine Learning Model for Overall Survival Prediction in Breast Cancer Using Clinical and Transcriptomic Data

**DOI:** 10.3390/biology14111539

**Published:** 2025-11-03

**Authors:** Mehmet Kivrak, Hatice Sevim Nalkiran, Oguzhan Kesen, Ihsan Nalkiran

**Affiliations:** 1 Department of Biostatistics and Medical Informatics, Faculty of Medicine, Recep Tayyip Erdogan University, 53020 Rize, Türkiye; mehmet.kivrak@erdogan.edu.tr; 2 Department of Medical Biology, Faculty of Medicine, Recep Tayyip Erdogan University, 53020 Rize, Türkiye; hatice.sevim@erdogan.edu.tr; 3 Department of Medical Oncology, Faculty of Medicine, Recep Tayyip Erdogan University, 53020 Rize, Türkiye; oguzhan.kesen@erdogan.edu.tr

**Keywords:** luminal A breast cancer, machine learning, gene expression, age, survival prediction, XGBoost

## Abstract

Breast cancer is the most common malignancy in women, with the Luminal A subtype generally associated with favorable survival. However, age and menopausal status may influence tumor biology and prognosis. To improve prediction beyond conventional models, we analyzed transcriptomic and clinical data from the METABRIC cohort. Patients with Luminal A breast cancer were stratified into premenopausal, postmenopausal–nongeriatric, and geriatric (≥70 years) groups. Differentially expressed genes (DEGs) were identified, and Boruta feature selection revealed 27 clinical and genomic variables. Random Forest, Logistic Regression, Multilayer Perceptron, and ensemble XGBoost models were trained with stratified 5-fold cross-validation, using SMOTE to correct class imbalance. Principal component analysis showed distinct clustering across age groups, while DEG analysis revealed 41 genes associated with age and survival. Key predictors included clinical variables (age, tumor size, NPI, radiotherapy) and molecular markers (ATM, HERC2, AKT2, FOXO3, CYP3A43). Among ML models, XGBoost demonstrated the highest performance (accuracy 98%, sensitivity 98%, specificity 97%, F1-score 0.99, AUC 0.86), outperforming other algorithms. These findings indicate that age-related transcriptomic changes impact survival in Luminal A breast cancer and that an ML-based integrative approach combining clinical and molecular variables provides superior prognostic accuracy, supporting its potential for clinical application.

## Simple Summary

Breast cancer is the most common cancer in women, and the Luminal A type is usually linked to better survival. However, age and menopause can affect how the disease behaves and how patients respond to treatment. In this study, we looked at both genetic information from tumors and clinical features such as age, tumor size, and treatments. Women with Luminal A breast cancer were divided into younger, older, and elderly groups. We found that gene activity differed between these groups and that some genes and clinical features were closely related to survival. By using computer-based learning methods, we created models that combined both genetic and clinical data. These models predicted survival more accurately than traditional methods. Our results suggest that, in future, considering both age-related genetic changes and clinical features may help doctors make better treatment decisions and improve outcomes for women with this type of breast cancer.

## 1. Introduction

Breast cancer is the most common malignancy among women worldwide and is characterized by a highly heterogeneous clinical course and molecular profile [1]. With increasing understanding of this heterogeneity, the importance of molecular subtypes in diagnosis and treatment has become more evident. Based on gene expression profiles, breast cancer is classified into luminal A, luminal B, HER2-positive, basal-like, and normal-like subtypes, each exhibiting distinct transcriptomic patterns. Consequently, identifying common biomarkers that encompass all subtypes remains a major challenge [2,3]. The luminal A subtype is characterized by high hormone receptor positivity, low proliferative activity, and relatively favorable survival outcomes [4,5]. However, accumulating evidence indicates that patients within this group do not exhibit uniform biological behavior. In particular, age, menopausal status, and tumor microenvironmental factors may significantly influence disease progression [6,7,8]. Understanding how these clinical and molecular factors interact is essential for improving prognostic precision and identifying age-specific biomarkers.

Breast cancer arises from a combination of genetic susceptibility, hormonal regulators, environmental exposures, and lifestyle factors. This multidimensional interplay is a key driver of tumor heterogeneity and clinical variability [9,10,11]. Clarifying these complex interactions not only contributes to prevention and early diagnosis but also provides opportunities to discover novel therapeutic targets. Age is especially critical for both tumor biology and therapeutic response. In geriatric patients, the tumor microenvironment may undergo distinct changes due to a weakened immune system, hormonal imbalances, and systemic physiological alterations [8,12,13,14,15]. As a result, tumors in older individuals may appear genetically indolent or, conversely, display resistant profiles. By contrast, breast cancers in younger patients often exhibit more aggressive courses with higher proliferation indices.

The menopausal transition further accentuates these biological differences, as declining estrogen levels affect hormone receptor expression and tumor responsiveness [16,17,18,19]. All these findings suggest that age and menopausal status shape luminal A breast cancer prognosis, yet it remains unclear how these differences manifest at the molecular level and how they ultimately affect survival outcomes [20,21,22,23]. Addressing this knowledge gap is essential for developing age-sensitive prognostic strategies.

In recent years, the expansion of big data resources and advances in information technology have accelerated the integration of machine learning (ML) into medical applications, yielding significant improvements in breast cancer diagnosis. For example, Liu et al. [24] achieved high diagnostic accuracy using ML models based on imaging data, while Nupa et al. [25] demonstrated that various classifier approaches achieve comparable performance across training and test sets. These studies collectively indicate that ML algorithms can robustly learn complex diagnostic features, providing a foundation for translational applications in oncology. Beyond imaging, artificial intelligence and ML have contributed to decoding genomic regulatory networks and facilitated the analysis and classification of large-scale clinical genomic data [26,27,28]. These methods are increasingly employed to identify biologically meaningful genes from extensive lists of differentially expressed genes (DEGs) [29].

In this study, we aimed to assess the impact of differentially expressed genes on overall survival (OS) in patients with luminal A breast cancer from the METABRIC dataset, divided into three age groups: premenopausal, postmenopausal non-geriatric, and geriatric (≥70 years). By integrating clinical and genomic variables through ML-based gene selection and multi-cohort validation, we developed an integrative prognostic model. This model incorporates age-specific expression patterns to enhance individualized risk assessment and holds significant potential to identify age-sensitive molecular prognostic factors, improve clinical decision support, and refine patient-level survival predictions. By combining molecular biology with advanced computational techniques, the present study aims to bridge the gap between biological heterogeneity and predictive modeling in luminal A breast cancer.

## 2. Material and Methods

### 2.1. Bioinformatic Processing of Microarray-Based Gene Expression Data

We used microarray-based gene expression data from the METABRIC (Molecular Taxonomy of Breast Cancer International Consortium) study, originally published by Curtis et al. [5]. This dataset includes genome-wide expression profiles from 1992 clinically annotated primary breast tumor samples, generated using the Illumina HumanHT-12 v3 Expression BeadChip platform. The METABRIC microarray-based gene expression data (Illumina, San Diego, CA, USA; HT-12 platform; originally deposited under EGA accession number EGAS00000000083, dataset EGAD00010000162) together with matched clinical annotations were obtained through cBioPortal (https://www.cbioportal.org/, accessed on 1 June 2025). For this study, patients were re-stratified based on clinical and molecular parameters relevant to our machine learning framework. After excluding cases with incomplete annotations, a subset of 679 patients with complete clinical and genomic data was retained for downstream analyses. Under this reclassification, patients were stratified into subgroups based on age (premenopausal, postmenopausal–non-geriatric, geriatric) and clinical outcome (alive, died of disease) (Table 1).

### 2.2. Data Preprocessing

During the data preprocessing stage, gene identifiers were matched against a reference database to enable automated conversion and functional annotation. Genes were filtered using the limma moderated t-statistics with Benjamini–Hochberg (BH) FDR ≤ 0.1 and |log2 fold change| ≥ 2. All filtering and integration steps were performed in R software (version 4.2.3), primarily using the dplyr package. The original METABRIC dataset comprised 1992 breast cancer cases. For this study, only patients with complete information on age, menopausal status, and survival outcomes within the Luminal A subtype were considered. After excluding samples with missing demographic or survival data, 679 patients remained eligible for reclassification based on age and clinical outcome (premenopausal, postmenopausal–non-geriatric, geriatric; alive vs. died of disease). Subsequent integration and preprocessing steps further excluded patients with incomplete morphological or treatment-related variables, yielding a final dataset of 458 patients with comprehensive demographic, clinical, and genomic information.

Initial inspection of gene expression distributions revealed skewness, which was corrected by applying a log2 transformation. This transformation preserved small values while reducing variance and had a particularly pronounced effect on downregulated genes. Following normalization and transformation, systematic differences in gene expression distributions were largely eliminated, ensuring comparability across samples. To assess potential batch effects, principal component analysis (PCA) was conducted. The results indicated that clustering was primarily explained by biological subgroups (age and survival outcome), with minimal technical variation. This confirmed that batch effects did not interfere with downstream analyses.

### 2.3. Differentially Expressed Gene (DEG) Analysis

In this study, breast cancer patients with the Luminal A subtype from the METABRIC dataset were stratified into three age groups: premenopausal, postmenopausal non-geriatric, and geriatric (≥70 years). DEGs were identified using linear models implemented in the limma package, with contrasts defined across age groups (geriatric vs. premenopausal; postmenopausal non-geriatric vs. premenopausal; geriatric vs. postmenopausal non-geriatric) and including survival status as a covariate. Genes were filtered using the limma moderated t-statistics with BH correction (FDR ≤ 0.1) and |log₂ fold change| ≥ 2. All statistical analyses were carried out in R software (version 4.2.3) using the limma package [30,31].

### 2.4. Principal Component Analysis (PCA)

Principal component analysis (PCA) was conducted to examine global variation in gene expression profiles and to reveal potential clustering among clinical subgroups. The transcriptomic data used in the analysis were obtained from the METABRIC (Molecular Taxonomy of Breast Cancer International Consortium) study and were profiled using the Illumina HumanHT-12 v3 platform [5]. During the preprocessing steps, gene identifiers were matched with a reference database, and low-variance genes were removed using interquartile range filtering. Log₂ transformation followed by normalization was then applied. Differential expression was subsequently defined as |log₂ fold change| ≥ 2 with BH adjusted FDR ≤ 0.1. After integration with clinical and demographic data, a total of 489 breast cancer–related genes and 458 patients with complete clinical annotation were included in downstream analyses. PCA was performed in R software (version 4.2.3) using the prcomp function on centered and scaled values, and visualizations were generated using the ggplot2 package.

### 2.5. Biostatistical Analysis

Breast cancer patients were classified into age groups (premenopausal, postmenopausal non-geriatric, and geriatric) and statistically evaluated for the survival status variable using clinical and demographic data. Continuous variables were presented as mean ± standard deviation ($\bar{X}$ ± SD), while categorical variables were expressed as frequency (percentage, %). The assumption of normality for continuous variables was assessed using the Shapiro–Wilk test, and the homogeneity of variances across groups was examined using the Levene test. For data meeting both normality and homogeneity assumptions, independent samples *t*-tests were used to compare group means, whereas associations between categorical variables were analyzed using the chi-square test. The assumption of normal distribution was tested using the Kolmogorov–Smirnov test. Differences between two groups in continuous variables were assessed with the independent samples t-test, whereas associations between categorical variables were examined using the chi-square test. All analyses were performed using Jamovi software (v2.4.6), and a significance level of *p* < 0.05 was considered for all tests.

### 2.6. Machine Learning

Machine learning is one of the most prominent and rapidly evolving fields in modern computer science. While learning is a natural human behavior, various methods have been developed to establish it as a fundamental capability for machines as well. These techniques have been successfully applied across a wide spectrum, including computer vision, predictive modeling, semantic analysis, natural language processing, knowledge extraction, object recognition and detection, classification, recommendation systems, text and document classification, image analysis, as well as medical diagnosis and prognostic modeling [32].

### 2.7. Prediction Model

In this study, Logistic Regression (LR), Multilayer Perceptron (MLP), Random Forest (RF), and Ensemble XGBoost (XGB) algorithms were employed to classify the survival status label variable in breast cancer. Logistic regression is a widely used method in clinical research for modeling binary outcomes and quantifies the independent effect of each variable through the odds ratio (OR) [33]. The Multilayer Perceptron (MLP) learns nonlinear relationships by employing an artificial neural network architecture composed of input, hidden, and output layers [34]. Random Forest (RF) enhances predictive performance and limits overfitting by constructing multiple decision trees through the random sampling (bagging) method [35]. XGBoost is an efficient implementation of gradient-boosted decision trees that prevents overfitting through regularization techniques and is widely applied in biomedical data analysis [36].

The dataset was randomly split into 70% training and 30% testing subsets, and to prevent data leakage, the Synthetic Minority Over-Sampling Technique (SMOTE) was strictly applied only to the training folds during stratified 5-fold cross-validation. For each iteration, oversampling was performed independently within the training partition of each fold, ensuring that validation folds remained untouched and reflected the original class distribution. The same principle was applied to the final evaluation: the independent test set was never oversampled and retained its natural imbalance (Alive = 96; Died = 42). This procedure ensured that the oversampling process influenced only model training and not model validation or testing, thereby maintaining the integrity of the performance estimates. The test set, however, was left unaltered to preserve the original distribution. SMOTE is a method designed to mitigate class imbalance by generating synthetic samples of the minority class based on feature similarity [37].

Feature selection is a critical step in bioinformatics studies for identifying potential biomarkers. In this study, the Boruta algorithm (Random Forest by Gini) was employed for feature selection, which was performed exclusively on the training set. All modeling and analysis steps were conducted using R software (version 4.2.3). The glm() function was applied for logistic regression, the randomForest package for RF, the nnet package for MLP, and the xgboost package for XGBoost. SMOTE was implemented using the DMwR package. Model performance was evaluated based on accuracy, sensitivity, specificity, F1-score, and AUC metrics, while hyperparameter optimization was carried out using the grid search method within the caret package.

## 3. Results

### 3.1. Global Gene Expression Variation and Subgroup Clustering Identified by PCA

**Figure 1:**
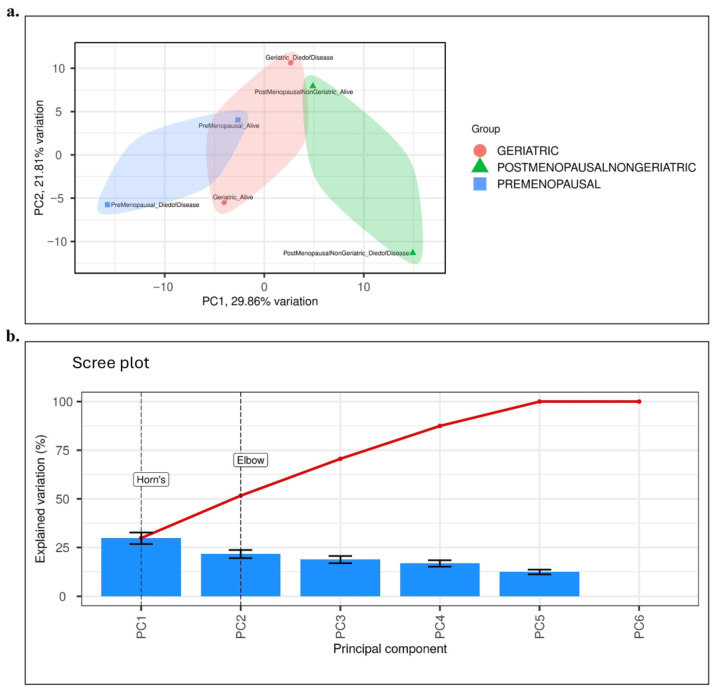
Results of Principal Component Analysis (PCA). (**a**) PCA score plot illustrating the distribution of patient samples according to clinical groups: Premenopausal (blue), Postmenopausal–non-geriatric (green), and Geriatric (red). Survival status is indicated by different symbols (circle: alive, triangle: died of disease). Ellipses represent the 95% confidence interval for each group. The first two principal components explain 29.9% (PC1) and 21.8% (PC2) of the total variance, respectively. (**b**) Scree plot analysis showing the contribution of principal components to the explained variance. Both Horn parallel analysis test and the elbow criterion confirmed that PC1 and PC2 represent the major sources of variance and should be considered in subsequent interpretations. Blue bars represent the explained variation (%) for individual PCs, while the red line indicates the cumulative variance across components.

The first two principal components, PC1 (29.9%) and PC2 (21.8%), together accounted for 51.7% of the total variance. This result was further validated by Horn parallel analysis test and the elbow criterion. Two-dimensional score plots were generated to visualize the distribution of patients. Clinical groups (premenopausal, postmenopausal–non-geriatric, and geriatric) were represented by colors, while survival status was indicated using distinct symbols. As shown in Figure 1a, the PCA distribution revealed clear separations among the clinical groups. The scree plot analysis presented in Figure 1b confirmed that PC1 and PC2 were the principal components to be evaluated in this study. Collectively, these findings demonstrate that transcriptomic variation based on clinical characteristics in breast cancer patients was reliably captured through PCA.

Batch effect assessment and correction were performed to evaluate potential technical variation in the METABRIC dataset. To assess whether technical factors could have influenced the results, batch effect analysis and correction were performed on the METABRIC dataset.. Following log₂ transformation and quantile normalization, PCA was performed on centered and scaled expression data to visualize the contribution of technical and biological factors to total variance. As shown in Figure 1a, PCA demonstrated that sample clustering was primarily explained by biological subgroups, namely age group and survival outcome, whereas batch-related variables showed no systematic separation. Because the METABRIC dataset was generated using a uniform Illumina HT-12 v3 platform and standardized processing pipelines, additional variance component analysis (PVCA) was not deemed necessary. No dominant batch effects were observed; therefore, no empirical correction was applied to the primary dataset. As a sensitivity analysis, downstream models were repeated using a ComBat-adjusted dataset, yielding almost identical outcomes (ΔAUC < 0.02; consistent gene rankings), confirming that batch effects did not influence the study conclusions.

### 3.2. Gene Expression Variations Among Geriatric, Postmenopausal, and Premenopausal Luminal A Patients

**Figure 2:**
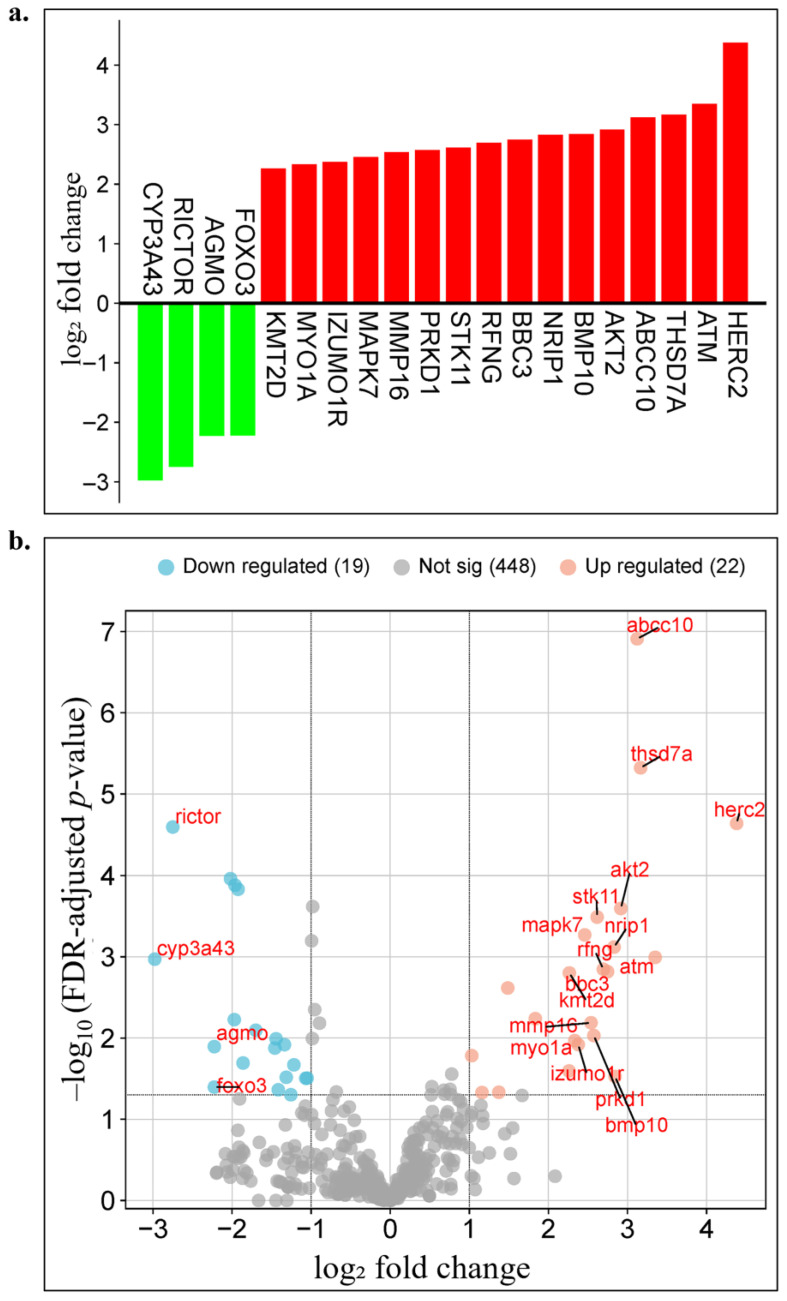
Visualization of age-related differential gene expression analysis results. (**a**) Bar plot displaying the log_2_ fold change values of differentially expressed genes between geriatric (≥70 years) and other age groups in Luminal A breast cancer patients. Red bars represent upregulated genes, whereas green bars indicate downregulated genes. (**b**) Volcano plot showing the distribution of all genes based on their log_2_ fold change and −log_10_(adjusted *p*-value) values. Upregulated genes are marked in red, downregulated genes in blue, and statistically nonsignificant genes in gray.

The genes showing significant differential expression are presented in Figure 2a,b. In Figure 2a, a bar plot displays genes ranked according to their log₂ fold change values, with upregulated genes highlighted in red (e.g., HERC2, ATM, THSD7A, AKT2, BBC3) and downregulated genes in green (e.g., CYP3A43, AGMO, RICTOR, FOXO3). Figure 2b illustrates a volcano plot in which all genes are positioned based on their log₂ fold change and −log_10_(adjusted *p*-value) values; upregulated genes are shown in red, downregulated in blue, and statistically nonsignificant genes in gray. According to the predefined statistical thresholds (|log_2_ fold change| ≥ 2 and FDR ≤ 0.1), a total of 41 genes exhibited significant differential expression, including 22 upregulated and 19 downregulated genes. The remaining 448 genes were not statistically significant. Several genes demonstrated marked expression changes with log₂ fold change values exceeding ±2. In particular, HERC2, ATM, THSD7A, and ABCC10 showed strong upregulation with |log_2_ fold change| > 3, whereas CYP3A43, RICTOR, AGMO, and FOXO3 displayed pronounced downregulation with |log_2_ fold change| < −2 (Figure 2, Table 2).

These findings demonstrate that distinct gene expression profiles emerge in Luminal A subtype breast cancer in an age-dependent manner. The identified DEGs provide an important resource for elucidating age-specific molecular mechanisms. These genes served as the primary data for constructing machine learning-based individualized prognostic models in the subsequent stage. Moreover, they may be further evaluated as potential biomarker candidates for the development of personalized prognostic models in future studies.

### 3.3. Evaluation of Target Gene Expression in Relation to Age Groups and Clinical Outcomes

**Figure 3:**
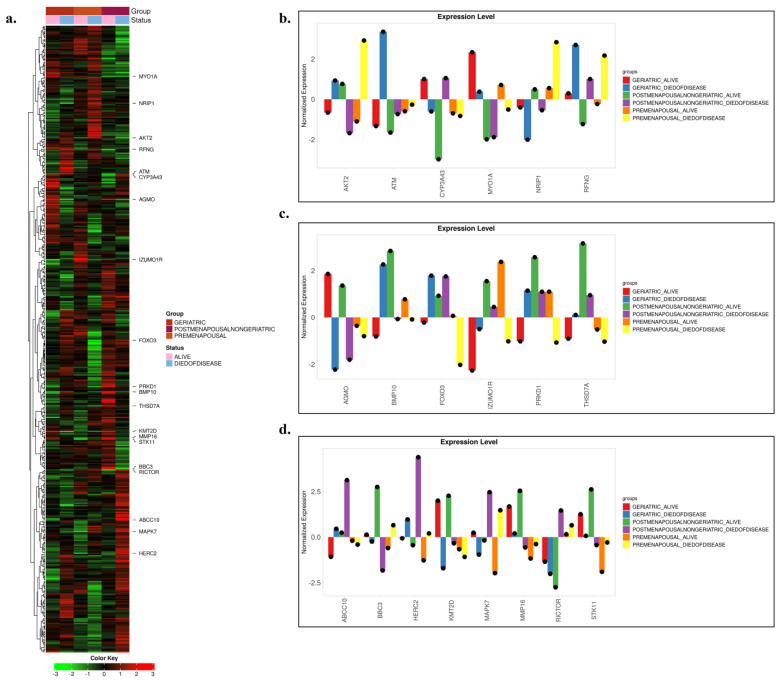
Differential gene expression across breast cancer patient subgroups. (**a**) Heatmap of genes showing statistically significant differences among the selected 489 genes. Patients are color-coded according to clinical groups (Geriatric, Postmenopausal–Non-geriatric, and Premenopausal), with survival status also indicated. The color scale (green → low expression, red → high expression) represents normalized log_2_-transformed expression levels. (**b**–**d**) Normalized expression levels of selected genes. The bar plots highlight distinct expression differences among patient subgroups and reveal potential biomarker candidates.

METABRIC transcriptome analyses (Figure 3a–d) were performed by jointly evaluating age groups (premenopausal, postmenopausal–non-geriatric, geriatric) and clinical outcomes (alive/died of disease). The findings revealed distinct expression patterns in many genes depending on age and clinical status, with the most pronounced separation observed in the postmenopausal–non-geriatric group. Overall assessment across the six subgroups indicated that certain genes consistently emerged as discriminative biomarkers along both the age and clinical outcome axes. Specifically, AKT2 showed strong upregulation in the premenopausal–died of disease group, reflecting a profile unique to young-age mortality. ATM was downregulated in the geriatric–alive group but upregulated in the geriatric–died of disease group, linking it to late-age mortality. CYP3A43 played a critical role in distinguishing survival versus mortality, particularly in the postmenopausal cohort. MYO1A was upregulated in association with survival at older ages, while also exhibiting a sharp alive–died of disease contrast in the premenopausal group. NRIP1 and RFNG displayed opposite directional patterns across age groups, offering age-specific discriminative profiles. Moreover, AGMO and KMT2D emerged as coordinated epigenetic and metabolic regulators reflecting survival–mortality divergence in older patients. BMP10 demonstrated strong upregulation associated with postmenopausal survival and geriatric mortality. FOXO3 presented an age-stage-sensitive pattern, showing downregulation in association with young-age mortality but upregulation with late-age mortality. IZUMO1R exhibited a dynamic, outcome-sensitive profile, being downregulated in association with survival in older patients, upregulated in younger and middle-aged groups, and downregulated again in premenopausal mortality. PRKD1 showed upregulation linked to survival in younger patients, downregulation with mortality, and upregulation observed again in geriatric mortality.

In addition, THSD7A displayed strong upregulation in postmenopausal survival, upregulation observed again in geriatric mortality, and consistent downregulation in younger patients. ABCC10 was markedly upregulated, particularly in postmenopausal mortality. BBC3 exhibited stage-dependent variation, with upregulation in postmenopausal survival and upregulation observed again in premenopausal mortality. HERC2 consistently showed downregulation across all alive groups and upregulation across all died of disease groups, indicating its robustness as an outcome-associated marker independent of age. MAPK7 was universally upregulated with mortality across all age strata, while showing age-dependent variation in survival, underscoring its strong clinical outcome sensitivity. MMP16 revealed upregulation with survival and downregulation with mortality in older patients, whereas in younger patients, it maintained a continuous downregulation pattern regardless of outcome. RICTOR demonstrated downregulation in older patients and outcome-independent upregulation in younger patients, indicating age-specific divergence. Finally, STK11 exhibited upregulation in geriatric alive groups but persistent downregulation in premenopausal patients, reflecting an age-dependent opposing expression profile.

**Figure 4:**
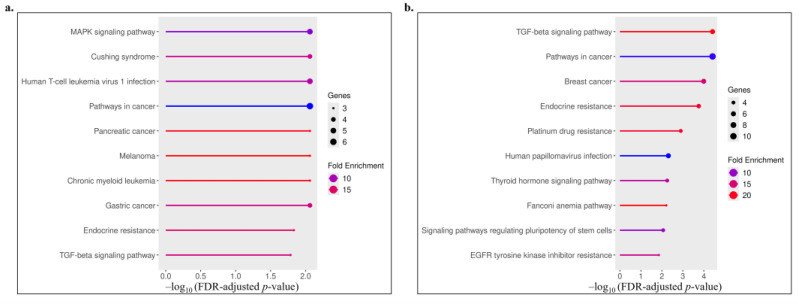
Visualization of the top enriched KEGG pathways for upregulated (**a**) and downregulated (**b**) genes. The x-axis represents the −log_10_ of the FDR-adjusted *p*-value, the dot size indicates the number of genes involved in each pathway, and the color gradient denotes fold enrichment.

KEGG pathway analysis revealed distinct enrichment patterns between upregulated and downregulated gene sets (Figure 4, Table 3). Upregulated genes were mainly associated with cancer-related and signaling pathways, including MAPK, TGF-β, and endocrine resistance, indicating activation of proliferative and stress-response mechanisms. In contrast, downregulated genes were enriched in pathways linked to drug resistance, hormone signaling, and stem cell regulation, such as platinum drug resistance, thyroid hormone, and pluripotency-related signaling. Overall, these results suggest that the treatment modulates key oncogenic and resistance-related pathways, reflecting both activation and suppression of critical molecular processes.

### 3.4. Statistical Analysis of Survival Status Across Geriatric, Premenopausal, and Postmenopausal Patients

As shown in Table 4, the mean age was significantly higher in the died of disease group compared to the alive group (64.2; t = −6.57, *p* < 0.001). Similarly, tumor size was larger in the died of disease group, and this difference was statistically significant (28.2; t = −5.17, *p* < 0.001). Survival time in months was markedly lower in the died of disease group, with the difference reaching a high level of statistical significance (103.0; t = 8.06, *p* < 0.001). In addition, Nottingham Prognostic Index (NPI) values were higher in the died of disease group compared to the alive group (4.01; t = −5.19, *p* < 0.001), indicating a strong prognostic association with mortality. 

According to the results presented in Table 5, significant associations were identified between certain clinical variables and survival status. A statistically significant relationship was observed between the type of breast surgery and survival status (χ^2^ = 30.0, *p* < 0.001). Similarly, radiotherapy was significantly associated with OS status (χ^2^ = 18.1, *p* < 0.001). In addition, primary tumor laterality showed a statistically significant relationship with OS status (χ^2^ = 5.51, *p* < 0.001). In contrast, no significant associations were found between survival status and other clinical or demographic variables.

These findings indicate that, in addition to continuous variables such as age, tumor size, survival time, and NPI, categorical variables including type of surgery, radiotherapy, and tumor laterality also serve as determining factors for survival status.

### 3.5. Machine Learning-Based and Ensemble Classification of Breast Cancer Survival Outcomes

**Figure 5:**
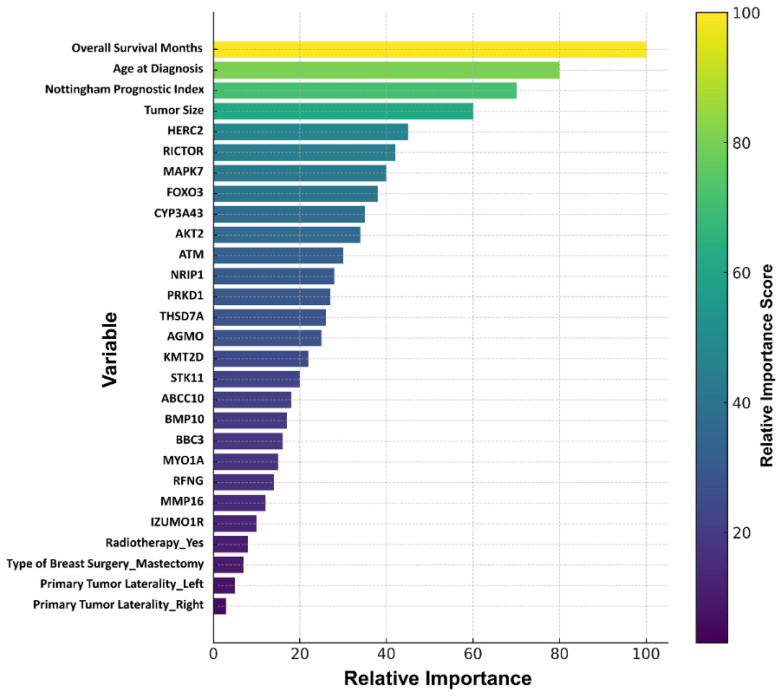
Clinical and gene variables identified by the Boruta algorithm.

The dataset consisted of a total of 458 patient records (313 alive, 145 died of disease) and was randomly split into 70% training (*n* = 320) and 30% testing (*n* = 138) subsets. To address class imbalance in the training set, SMOTE was applied, while the original distribution of the test set was preserved without any modifications. This ensured that the predictive performance of all models was evaluated on the same test set, reflecting the true data distribution. During the data partitioning stage, 30% of the data were reserved exclusively for testing, while the remaining 70% underwent stratified 5-fold cross-validation. This approach enabled the test set to serve as an independent validation cohort, allowing the assessment of model performance on previously unseen data. Statistically significant clinical and demographic variables, together with target genes identified from the DEG analysis (a total of 27 variables, Figure 5), were evaluated using the Boruta algorithm. The analysis identified variables with relative importance values ≥ 20%. The highest contributions were observed for Months (100.0), Age at diagnosis (68.2), Nottingham Prognostic Index (58.1), and Tumor size (53.1). At the gene level, the most prominent biomarkers included HERC2 (40.9), RICTOR (31.5), MAPK7 (30.5), FOXO3 (27.6), CYP3A43 (26.8), AKT2 (26.6), ATM (26.2), NRIP1 (26.1), PRKD1 (24.6), THSD7A (23.5), AGMO (23.1), KMT2D (22.7), and STK11 (20.1) (Figure 5).

The final set of variables was used to train baseline RF, LR, MLP, and Ensemble XGBoost models, and their predictive performance was compared on the test set using ROC curves. Overfitting and underfitting were assessed based on error rates calculated during the training and testing processes. In addition, grid search optimization was applied to identify the hyperparameters that yielded the highest predictive performance for each model (Table 6).

**Figure 6:**
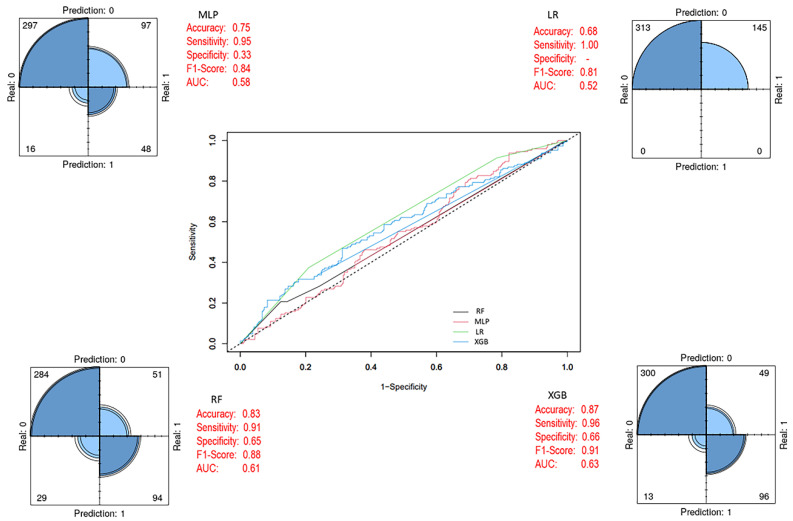
Prediction models with imbalanced class distribution (Before SMOTE). In the ROC curve, each line represents a different model: blue for XGB, green for LR, black for RF, and pink for MLP. The diagonal dashed line indicates random classification performance.

As shown in Figure 6, when trained on the imbalanced training set, the XGB ensemble model achieved higher accuracy, specificity, F1-score, and AUC values compared to the other three models.

**Figure 7:**
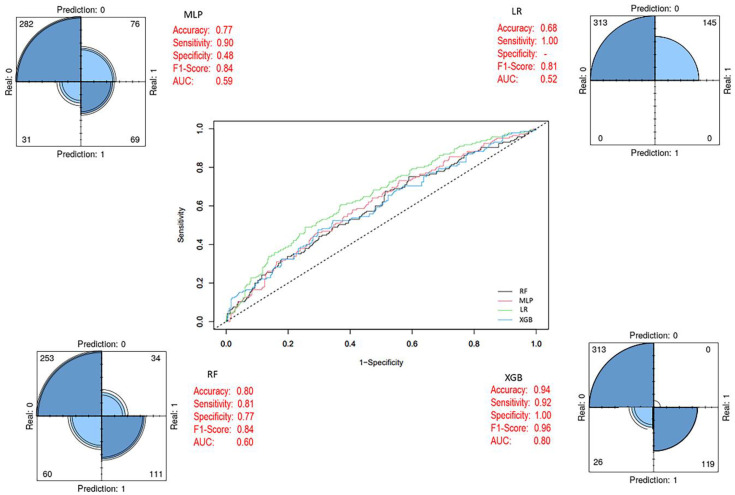
Prediction models with balanced class distribution (After SMOTE). In the ROC curve, each line represents a different model: blue for XGB, green for LR, black for RF, and pink for MLP. The diagonal dashed line indicates random classification performance.

**Figure 8:**
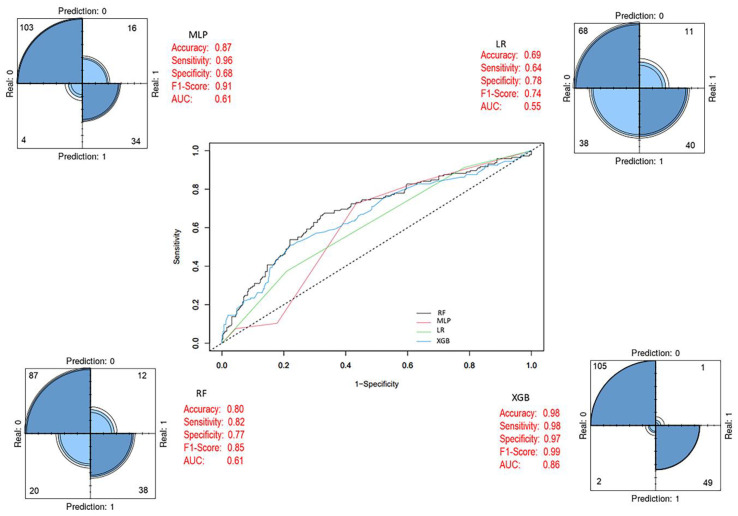
Final prediction models after feature selection (Last Model). In the ROC curve, each line represents a different model: blue for XGB, green for LR, black for RF, and pink for MLP. The diagonal dashed line indicates random classification performance.

By applying the SMOTE method, class imbalance in the training set was corrected, resulting in a balanced dataset comprising 434 patient samples (219 alive and 215 died of disease). Using this dataset, RF, LR, MLP, and XGB models were developed, with the same 27 variables included as input vectors, consistent with the baseline model. After eliminating class imbalance, the XGB ensemble model achieved the highest performance across evaluation metrics compared to the other models. In addition, the performance metrics of the baseline classifiers also showed significant improvements once class balance was established (Figure 7). After applying the SMOTE algorithm, the training dataset became balanced (Alive = 219; Died = 215), ensuring that model learning was not biased toward the majority class. All models were trained on this balanced dataset, whereas evaluation was performed on the original, independent test set (Alive = 96; Died = 42) to preserve the real-world distribution. As presented in Figure 7, balancing substantially improved the internal cross-validation performance of all classifiers, particularly enhancing the minority-class recall. The final XGBoost model (Figure 8) was trained on the SMOTE-balanced training set but evaluated on the imbalanced test cohort. This approach allowed the model to learn equally from both classes while maintaining an unbiased external evaluation. Decision thresholds were optimized using the Youden J statistic on validation folds. The final model achieved balanced accuracy = 0.97 (95% CI 0.93–0.99), sensitivity = 0.98 (95% CI 0.94–1.00), specificity = 0.97 (95% CI 0.92–0.99), and PR-AUC = 0.91. Calibration analysis using the Brier score (0.038) confirmed strong agreement between predicted probabilities and observed outcomes (Table S1). The corresponding confusion matrix showed only minor misclassifications near the decision boundary (Figure S1). Together, these results confirm that the high accuracy values reported in Table 7 and visualized in Figure 5, Figure 6 and Figure 7 are not artifacts of class imbalance or thresholding, but reflect a robust and well-calibrated discriminative performance.

The predictive performance of both models was evaluated on the test set, and the optimized model obtained after feature selection was compared with the baseline prediction models and the SMOTE-adjusted models. The 27 variables used during the training process were ranked by their importance for breast cancer OS status using the Boruta algorithm, resulting in the final selection of 17 variables. Of these, 4 were clinical variables and 13 were derived from mRNA expression data. As shown in Figure 8, notable improvements in performance metrics were observed after feature selection. During the test phase, the XGB model achieved the highest performance, with an accuracy of 98%, sensitivity of 98%, specificity of 97%, F1-score of 0.99, and an AUC of 0.86. Detailed performance metrics of the model are presented in Table 7.

## 4. Discussion

Breast cancer is the most common malignancy among women and one of the leading causes of cancer-related mortality [38]. Prognosis is influenced by multiple factors, including molecular subtype, pathological stage, and genetic characteristics [39,40]. Among these factors, age emerges as an important determinant [41,42]. Several retrospective cohort analyses have investigated biomarker differences in premenopausal and postmenopausal tumors, demonstrating that age remains an independent risk factor even after adjusting for stage, treatment, or tumor characteristics [43]. However, the association between age and survival has not been universally consistent [44,45]. Therefore, risk models based solely on clinicopathological indicators may be insufficient, while the integration of molecular markers appears critical for achieving more reliable and personalized predictions [46]. In this context, incorporating gene expression profiles into clinical prognostic models may contribute to a better understanding of age-related biological differences.

In recent years, the clinical use of gene expression profiling (GEP) analyses has increased, demonstrating predictive value not only in defining molecular subtypes but also in late recurrences [47,48,49,50,51,52]. GEP analyses have been found particularly useful in predicting prognosis and chemotherapy response in patients with early-stage HER2-positive and HER2-negative breast cancer [53,54,55]. Nevertheless, the use of GEP in elderly patient populations remains limited, and its clinical utility continues to be uncertain [56,57]. These insights provide an important foundation for developing machine learning-based predictive models that combine clinical and molecular data for improved prognostication.

In our study, clinical and transcriptomic data were integrated across age groups in Luminal A breast cancer patients, and ML-based prediction models were developed. Among the algorithms applied, the XGBoost model achieved the highest performance on the independent test set in terms of accuracy, sensitivity, specificity, F1-score, and AUC. This finding confirms the superiority of ensemble-based boosting methods in capturing complex nonlinear relationships and handling high-dimensional biological data [36,58]. Class imbalance in the dataset was addressed using the SMOTE method, which markedly improved predictive accuracy, particularly within the mortality group. These results suggest that modern data processing approaches can provide outcomes more closely aligned with clinical applications [59,60,61]. Feature selection was performed using the Boruta algorithm, which revealed that both clinical parameters (e.g., age, tumor size, NPI, radiotherapy) and transcriptomic biomarkers (e.g., ATM, HERC2, AKT2, CYP3A43, FOXO3) provided strong prognostic contributions. This integrative approach demonstrated superior performance compared to models relying solely on clinicopathological data [62,63].

Although LR is simple and interpretable, it showed low AUC (0.55) and limited accuracy (0.69) on the test set [62]. The RF algorithm was able to partially capture nonlinear relationships; however, its AUC values remained low on the test set (0.61). Due to its sensitivity to class imbalance, the performance improvement of RF was limited even after applying SMOTE. While RF is strong in terms of feature selection and variable importance ranking, its predictive performance has been reported to be weaker than that of XGBoost, consistent with findings in the literature [64].

Although the MLP model achieved a high accuracy (0.87) and F1-score (0.91), its AUC value (0.61) remained limited. This indicates that, while it classified the alive group effectively, its discriminative power for the died of disease group was weaker [65,66]. In contrast, XGBoost outperformed all other models across evaluation metrics, achieving 98% accuracy, 98% sensitivity, 97% specificity, an F1-score of 0.99, and an AUC of 0.86, establishing it as the most reliable model [36]. This superiority reflects the capacity of boosting algorithms to capture critical distinctions within high-dimensional and heterogeneous biological data. However, for clinical integration, it is important to complement such models with explainable artificial intelligence methods [67]. Furthermore, these exceptionally high values should be interpreted with caution due to the limited size of the test set (*n* = 138) and the absence of an external validation cohort. Validation in larger and independent cohorts will be required to confirm the real-world performance of the model.

At the molecular level, the genes HERC2 and ATM, both involved in DNA damage response and repair, emerged as key findings. HERC2 has been shown to play critical roles in DNA repair and to contribute to the maintenance of genomic integrity by regulating BRCA1 protein stability [68,69]. Although the precise role of HERC2 in breast cancer pathogenesis has not yet been fully elucidated, it has been reported as a critical target for further investigation [70]. ATM, on the other hand, has been identified as an independent prognostic factor associated with poorer metastasis-free survival [71]. Although rarely examined in large cohorts, suppression of ATM expression at both the mRNA and protein levels has been linked to adverse prognosis [72,73,74,75]. Conversely, some studies have reported increased ATM expression in ER-negative tumors [76]. Our findings suggest that these genes may be associated with age-related survival differences in the Luminal A subtype of breast cancer. Consistent with previous literature, our pathway-level analysis supports that these biomarkers participate in key oncogenic and signaling mechanisms relevant to breast cancer progression and prognosis. Comparable computational and transcriptomic approaches have also been successfully employed to identify prognostic and diagnostic biomarkers in breast cancer, reinforcing the biological validity and translational potential of our findings [77,78].

From a signaling perspective, RICTOR and AKT2 are of particular importance. RICTOR expression has been reported to be upregulated in invasive and high-grade breast cancers, supporting Akt-mediated survival, while loss of mTORC2 has been shown to enhance apoptosis [79]. Zhang et al. also confirmed RICTOR overexpression in human breast tumors [80]. Moreover, RICTOR expression has been found to be higher compared to normal tissues, inversely correlated with the Nottingham Prognostic Index and tumor grade, and associated with longer survival [81]. Our results extend these observations by showing that RICTOR expression diverges according to patient age, with downregulation in older patients and outcome-independent upregulation in younger patients. AKT2, on the other hand, plays a critical role in both tumor development and the reprogramming of glucose metabolism, and it has been shown to be overexpressed in multiple cancer types, including breast cancer [82,83]. Genes such as MAPK7, FOXO3, and CYP3A43 also hold prognostic significance. Suppression of MAPK7 has been shown to increase E-cadherin expression, thereby reducing migration and metastasis in breast cancer cells; in animal models, it regulates tumor growth and has been linked to metastatic risk in clinical studies of breast cancer [84,85,86,87]. Reduced FOXO3 mRNA expression has been associated with larger tumor size and advanced stages, while activation of this gene has been reported to suppress proliferation [88,89,90]. CYP3A43 polymorphisms, meanwhile, have been identified as potential biomarkers for breast cancer prognosis [91]. In addition, several other genes emerged as noteworthy in our study, including NRIP1, PRKD1, THSD7A, AGMO, KMT2D, and STK11. Suppression of NRIP1 has been reported to inhibit tumor growth, while expression changes in Luminal A tumors may hold clinical relevance [92,93]. PRKD1 shows high expression in normal ductal epithelium but is downregulated in invasive tumors, suggesting its potential as a marker for the invasive phenotype [94,95,96]. THSD7A is notable for its high expression frequency [97], whereas the role of AGMO in breast cancer remains insufficiently elucidated in the literature. Increased expression of KMT2D has been associated with poor prognosis [98]. STK11, on the other hand, functions as a tumor suppressor gene, and its reduced expression has been linked to higher histological grade, lymph node metastasis, and worse survival outcomes [99,100,101]. Furthermore, STK11 has been shown to act as a co-activator in estrogen receptor (ERα) signaling and to play a role in transcriptional regulation through its interaction with FOXO3 [102,103].

In conclusion, our findings reveal both universal mortality markers, such as HERC2 and MAPK7, and age-specific divergences exemplified by AKT2, FOXO3, and RICTOR, emphasizing the importance of integrating clinical variables with transcriptomic signatures to refine risk stratification in Luminal A breast cancer across different age groups. By combining advanced machine learning approaches with biological insights, we demonstrated that ensemble-based models such as XGBoost can yield robust predictive performance, while also uncovering key molecular drivers with potential clinical relevance. However, the absence of external validation and the relatively small test cohort highlight the need for confirmation in larger, independent datasets. Future studies should focus on validating these predictive frameworks, exploring explainable artificial intelligence strategies, and translating identified biomarkers into clinically actionable tools. Such efforts will ultimately contribute to more precise, age-tailored prognostic assessments and facilitate the development of personalized therapeutic strategies in breast cancer management.

Finally, several limitations of this study should be acknowledged. Although extensive data preprocessing and feature selection were implemented, the potential for minor information leakage cannot be entirely excluded, particularly if survival-associated variables indirectly influenced model training. In addition, treatment-related variables may have been subject to confounding by indication, as therapy allocation often correlates with disease stage and patient age. The relatively modest size of the independent test set and the absence of an external validation cohort also limit the generalizability of the results.

Future studies should address these limitations by incorporating survival-based modeling approaches, such as Cox proportional hazards, random survival forests, or gradient-boosting survival models, to better capture temporal risk dynamics. Furthermore, the integration of explainable artificial intelligence (XAI) tools, including SHAP or LIME, could enhance model interpretability and facilitate clinical translation. Validation in larger, multi-institutional cohorts will be essential to confirm the robustness and real-world utility of the proposed predictive framework.

## 5. Conclusions

This study emphasizes the importance of considering age-related biological differences in survival prediction for Luminal A breast cancer. Analyses conducted using the METABRIC dataset demonstrated that integrating gene expression profiles with classical clinical variables achieved higher predictive accuracy compared to clinicopathological models alone. Among the applied machine learning approaches, the XGBoost model exhibited the highest performance; in addition to clinical factors such as age, tumor size, NPI, and radiotherapy, molecular biomarkers including ATM, HERC2, AKT2, FOXO3, and CYP3A43 provided critical contributions to survival prediction. The findings indicate that age-specific biological differences should not be overlooked in prognostic modeling studies of breast cancer and that machine learning-based approaches have the potential to enhance personalized risk assessments. Although the XGBoost model achieved very high predictive accuracy (98%), such values should be interpreted with caution. Despite rigorous cross-validation, balanced evaluation, and calibration analyses, the relatively limited test cohort (*n* = 138) may contribute to optimistic estimates. Therefore, the reported metrics represent a proof-of-concept demonstration that requires validation in larger, independent datasets.

## Figures and Tables

**Table 1:** Classification of the METABRIC cohort according to age groups and clinical outcomes.

Sample Name	Group	Status
Geriatric_Alive	Geriatric	Alive
Geriatric_Died of Disease	Geriatric	Died of Disease
PreMenopausal_Alive	PreMenopausal	Alive
PreMenopausal_Died of Disease	PreMenopausal	Died of Disease
PostMenopausalNonGeriatric_Alive	PostMenopausalNonGeriatric	Alive
PostMenopausalNonGeriatric_Died of Disease	PostMenopausalNonGeriatric	Died of Disease

**Table 2:** Genes identified as significant in the differential gene expression analysis.

Gene Name	Log_2_ Fold Change	BH-Adjusted *p*-Value (FDR)	Direction
*CYP3A43*	−2.9775	0.040146	Down
*RICTOR*	−2.7493	0.000148	Down
*AGMO*	−2.2243	2.55 × 10⁻⁵	Down
*FOXO3*	−2.2235	0.000132	Down
*KMT2D*	2.2647	0.020281	Up
*MYO1A*	2.3325	0.000109	Up
*IZUMO1R*	2.3771	0.005937	Up
*MAPK7*	2.4586	0.008039	Up
*MMP16*	2.5389	0.013266	Up
*PRKD1*	2.5745	0.010218	Up
*STK11*	2.6159	0.043386	Up
*RFNG*	2.6941	0.012076	Up
*BBC3*	2.7481	0.030447	Up
*NRIP1*	2.8304	0.021395	Up
*BMP10*	2.8427	0.049004	Up
*AKT2*	2.9156	0.016484	Up
*ABCC10*	3.1221	0.046946	Up
*THSD7A*	3.166	0.046532	Up
*ATM*	3.3504	0.002421	Up
*HERC2*	4.38	0.005774	Up

**Table 3:** The top enriched KEGG pathways identified from upregulated and downregulated gene sets.

Group	FDR	Fold Enriched	Pathway
Upregulated	8.66 × 10⁻³	8.032407407	MAPK signaling pathway
Upregulated	8.66 × 10⁻³	12.51803752	Cushing syndrome
Upregulated	8.66 × 10⁻³	10.80592925	Human T-cell leukemia virus 1 infection
Upregulated	8.66 × 10⁻³	5.466288595	Pathways in cancer
Upregulated	8.66 × 10⁻³	18.77705628	Pancreatic cancer
Upregulated	8.66 × 10⁻³	19.80593607	Melanoma
Upregulated	8.66 × 10⁻³	19.02412281	Chronic myeloid leukemia
Upregulated	8.66 × 10⁻³	12.93810589	Gastric cancer
Upregulated	1.47 × 10⁻²	15.06076389	Endocrine resistance
Upregulated	1.64 × 10⁻²	13.51246106	TGF-beta signaling pathway
Downregulated	3.83 × 10⁻⁵	20.26869159	TGF-beta signaling pathway
Downregulated	3.83 × 10⁻⁵	6.832860744	Pathways in cancer
Downregulated	1.01 × 10⁻⁴	14.65371622	Breast cancer
Downregulated	1.75 × 10⁻⁴	18.82595486	Endocrine resistance
Downregulated	1.25 × 10⁻³	19.27777778	Platinum drug resistance
Downregulated	4.88 × 10⁻³	6.532379518	Human papillomavirus infection
Downregulated	5.55 × 10⁻³	12.04861111	Thyroid hormone signaling pathway
Downregulated	6.11 × 10⁻³	20.85336538	Fanconi anemia pathway
Downregulated	8.63 × 10⁻³	10.04050926	Signaling pathways regulating pluripotency of stem cells
Downregulated	1.39 × 10⁻²	13.72626582	EGFR tyrosine kinase inhibitor resistance

**Table 4:** Biostatistical Analysis Results of Continuous Variables.

$\mathrm{Group}\;(\bar{X}$ ± S_d_)	Alive (*n* = 313)	Died of Disease (*n*= 145)	Test Statistics	*p*-Value *
Age	56.6 ± 11.0	64.2 ± 12.7	−6.57	<0.001
Tumor Size	21.9 ± 9.9	28.2 ± 16.0	−5.17	<0.001
Months	160.1 ± 72.6	103.0 ± 66.0	8.06	<0.001
Nottingham Prognostic Index	3.46 ± 0.9	4.01 ± 1.2	−5.19	<0.001

$\bar{X\;}$= Mean, S_d_ = Standard Deviation, * = Independent Sample *t* Test.

**Table 5:** Biostatistical Analysis Results of Categorical Variables.

Group (Count (%))	Alive (*n* = 313)	Died of Disease (145)	Test Statistics	*p*-Value *
**Type of breast surgery**				
Mastectomy	143 (45.7)	106 (73.1)	30.0	<0.001
Breast conserving surgery	170 (54.3)	39 (26.9)		
**Chemotherapy**				
Yes	34 (10.9)	17 (11.7)	0.0743	0.785
No	279 (89.1)	128 (88.3)		
**Radiotherapy**				
Yes	204 (65.2)	64 (44.1)	18.1	<0.001
No	109 (34.8)	81 (55.9)		
**Histological grade high**				
Yes	104 (33.2)	34 (23.4)	0.89	0.123
No	209 (66.8)	111 (76.6)		
**Histological grade low**				
Yes	53 (16.9)	37 (25.5)	0.95	0.095
No	260 (83.1)	108 (74.5)		
**Histopathological type**				
Invasive Ductal Carcinoma (IDC)	237 (75.7)	112 (77.2)		
Invasive Lobular Carcinoma (ILC)	22 (7.0)	13(9.0)	0.025	0.542
Mixed tumor IDC + ILC	54 (17.3)	20 (13.8)		
**Primary tumor laterality**				
Right	157 (53.2)	59 (41.3)	5.51	0.019
Left	138 (46.8)	84 (58.7)		
**Cellularity**				
High	283 (90.4)	134 (93.7)	0.012	0.689
Low	30 (9.6)	9 (6.3)		
**HER2 loss**				
Yes	14 (4.5)	7 (4.8)		
No	299 (95.5)	138 (95.2)	0.081	0.793
**Tumor stage**				
Stage I	150 (47.9)	54 (37.2)		
Stage II	151 (48.2)	78 (53.8)	1.12	0.056
Stage III	9 (2.9)	11 (7.6)		
Stage IV	3 (1.0)	2 (1.4)		

* = Independent Sample Chi-Square Test.

**Table 6:** Hyperparameter Optimizations of the Models.

Model	Parameter	Values
RF	Bootstrap	(True, False)
	oob_score	(True, False)
	max_depth	3, 4, 5, 6, 7
	n_estimators	50, 100, 150, 200, 250
	min_samples_split	2, 3, 4, 5
	max_leaf_nodes	None, 2, 3, 4
MLP	hidden_layer_sizes	(10, 10), (15, 15), (20, 10), (20, 15)
	Activation	tanh, relu
	learning_rate	0.01, 0.001
LR	C	0.5, 1, 2, 3, 4, 5, 6
	Penalty	L1, L2, elasticnet
	Solver	Newton-cg, lbfgs, saga
	max_iter	50, 100, 200
	class_weight	Balanced, None
XGB	min_child_weight	1, 3, 5, 7, 10
	Gamma	1, 3, 5, 7, 10
	colsample_bytree	0.4, 0.5, 0.6
	reg_alpha	0, 0.2, 0.3
	max_depth	4, 5, 6
	Subsample	0.6, 0.7, 0.8
	n_estimators	100, 200, 300, 400, 500
	learning_rate	0.1, 0.01

**Table 7:** Performance Metrics of Baseline and Ensemble Models.

Model	Performance Metrics				
Random Forest	Accuracy	Sensitivity	Specificity	F1 Score	AUC
Before SMOTE (Training)	0.83	0.91	0.65	0.88	0.61
After SMOTE (Training)	0.80	0.81	0.77	0.84	0.60
Last Model (Testing)	0.80	0.82	0.77	0.85	0.61
Logistic Regression					
Before SMOTE (Training)	0.68	1.00	-	0.81	0.52
After SMOTE (Training)	0.68	1.00	-	0.81	0.52
Last Model (Testing)	0.69	0.64	0.78	0.74	0.55
Multilayer Perceptron					
Before SMOTE (Training)	0.75	0.95	0.33	0.84	0.58
After SMOTE (Training)	0.77	0.90	0.48	0.84	0.59
Last Model (Testing)	0.87	0.96	0.68	0.91	0.61
XGBoost					
Before SMOTE (Training)	0.87	0.96	0.66	0.91	0.63
After SMOTE (Training)	0.94	0.92	1.00	0.96	0.80
Last Model (Testing)	**0.98**	**0.98**	**0.97**	**0.99**	**0.86**

## Data Availability

The data are available from the authors on reasonable request.

## Supplementary Materials

The following supporting information can be downloaded at: https://www.mdpi.com/article/10.3390/biology14111539/s1, Table S1, presenting detailed performance metrics and calibration results (balanced accuracy, sensitivity, specificity, PR-AUC, and Brier score), and Figure S1, showing the confusion matrix for decision thresholds optimized using Youden’s J statistic.
