# Supplementary material for: Integrative Machine Learning Model for Overall Survival Prediction in Breast Cancer Using Clinical and Transcriptomic Data
Source: Biology (Basel). 2025 Nov 3;14(11):1539. doi: 10.3390/biology14111539 (PMC12650249; doi:10.3390/biology14111539)
Supplement: Supplementary file 1 [file biology-14-01539-s001.zip › biology-3923860-supplementary.pdf]

Table S1. Decision thresholds were optimized via Youden's J statistic

Metric	Value	95 % CI
Decision threshold	Optimized via *Youden's J* on validation folds	—
Balanced Accuracy	0.97	0.93 – 0.99
Sensitivity (Recall)	0.98	0.94 – 1.00
Specificity	0.97	0.92 – 0.99
PR-AUC	0.91	—
Brier Score	0.038	—

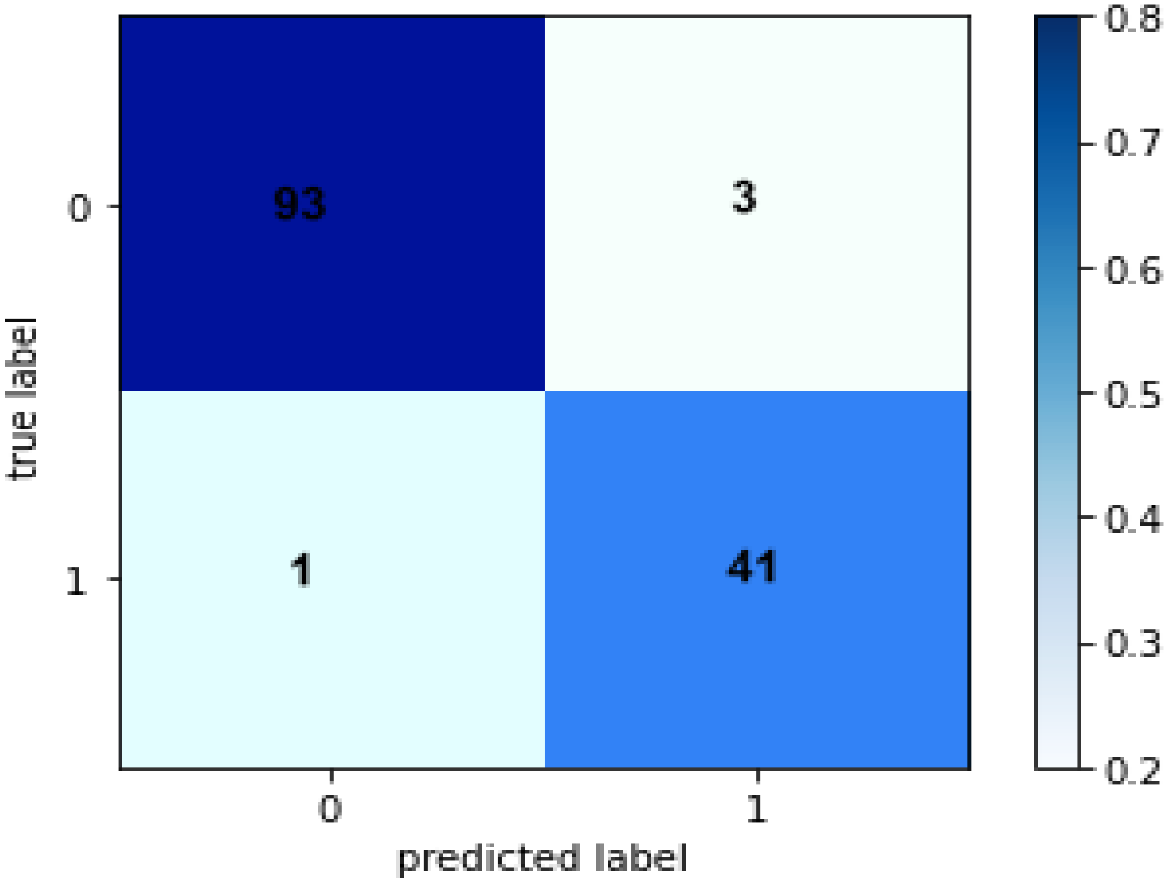

Figure S1. Confusion matrix for decision thresholds
